# The Prevalence and Incidence of Glaucoma in Denmark in a Fifteen Year Period: A Nationwide Study

**DOI:** 10.1371/journal.pone.0132048

**Published:** 2015-07-16

**Authors:** Miriam Kolko, Anna Horwitz, John Thygesen, Jørgen Jeppesen, Christian Torp-Pedersen

**Affiliations:** 1 Department of Neuroscience and Pharmacology, University of Copenhagen, Copenhagen, Denmark; 2 Department of Ophthalmology, Roskilde University Hospital, Roskilde, Denmark; 3 Center for Healthy Aging, University of Copenhagen, Copenhagen, Denmark; 4 Department of Ophthalmology, Copenhagen University Hospital Glostrup, Glostrup, Denmark; 5 Department of Cardiology, Copenhagen University Hospital Glostrup, Glostrup, Denmark; 6 Department of Health, Science and Technology, Aalborg University Hospital, Aalborg, Denmark; Massachusetts Eye & Ear Infirmary, Harvard Medical School, UNITED STATES

## Abstract

**Purpose:**

The purpose of the present study was to describe the prevalence, incidence and geographic variation of glaucoma in Denmark in the period from 1996 to 2011. Moreover, the aim was to identify the treatment patterns of glaucoma within the studied period.

**Methods:**

All Danish citizens were included throughout the study period. The National Prescription Registry was used to identify all claimed prescriptions for glaucoma medication.

**Results:**

A total of 116,592 incident glaucoma patients were identified. Average age at onset was 66 years (range: 0–105 years), 55% were women. The prevalence of glaucoma increased from 0.79% to 1.72% during the investigated period. In 2011 glaucoma affected 3.76% of the population above 50 years and 10% in patients above 80 years. The age-specific incidence rate of glaucoma seemed to be constant and the increasing prevalence was primarily attributed to an aging population. We found the highest prevalence of glaucoma in the capital region of Denmark. Within the studied period the use of prostaglandin analogs and combination drugs increased, whereas the use of β-blockers, carbon anhydrase inhibitors and parasympathomimetic drugs decreased (p<0.001). Finally, the use of α2-adrenergic agonists remained unchanged. A total of 75% of the patients were treated with two or more glaucoma medications.

**Conclusions:**

Over all, the present study is the first to assess the frequency and the development of glaucoma in Denmark over a 15-year period. We find that glaucoma affects a little less than 2% of the total population and increases with age to reach a prevalence of more than 10% amongst people above 80 years. Generally, the present study is the largest nation-wide study ever made and must be a close-to-real-life-picture of the utilization of glaucoma medication on a national scale. Our findings confirm other recent estimations on an increasing burden of glaucoma globally.

## Introduction

Glaucoma is one of the leading causes of blindness worldwide. According to The World Health Organization glaucoma accounted for 2% of visual impairment and 8% of global blindness in 2010, and the number of glaucoma patients is estimated to increase due to an aging population[1–3]. Generally, glaucoma refers to a group of eye conditions, which cause progressive damage to the optic nerve. If untreated, glaucoma will lead to permanent vision loss starting with unnoticeable blind spots at the edges of the field of vision, progressing to tunnel vision, and in worst case lead to blindness. The classification of glaucoma relies on the appearance and obstruction of the drainage pathway. In open angle glaucoma (OAG) the drainage pathway appears normal and in angle-closure glaucoma (ACG) the drainage pathway is obstructed. The pathogenesis of glaucoma is multifactorial. However, the intra ocular pressure (IOP) is the most evident risk factor for glaucomatous damage and to date IOP lowering drugs remain the only clinically validated treatment of glaucoma[4,5]. Historically, the first person to link IOP to the glaucoma was the English ophthalmologist, Richard Bannister, who described the phenomenon in 1622[6]. In1862 it was recognized that the extract from the calabar bean could counteract mydriasis, and in 1876 pilocarpin was introduced. The development of pressure lowering drugs accelerated in 1978 with the approval of Timolol[6]. In addition to glaucoma medication, glaucoma surgery has been performed since 1856 when Albrecht von Graefe introduced iridectomy. Hundred years later the most common glaucoma surgical procedure, trabeculectomy, was developed[7,8]. In spite of the development of more surgical procedures the far most predominant treatment of glaucoma is pressure lowering eye drops.

The prevalence of glaucoma has been reported in numerous studies and significant ethnic and geographic differences have been found. In spite of the unique possibility to estimate the prevalence of glaucoma in the entire population in Denmark only a few epidemiologic studies have been performed. To our knowledge the first study to estimate the number of glaucoma patients in Denmark was performed in 1989. This study was based on a national consumption of anti-glaucomatous drugs and revealed a prevalence of 0.76% in the age group above 40 years[9]. A follow up of this study was performed in 2000 based on a questionnaire survey among private practice ophthalmologists in Denmark. In this study the prevalence in Denmark was estimated to 0.65% in the entire population[10]. A recent study focused on the annual costs of glaucoma in Denmark and revealed an incidence rate of 1.2 per 1000 adults in the period from 2002 to 2007[11]. To our knowledge no previous study has benefited from a complete data sample from an entire population to estimate the incidence and prevalence of glaucoma.

Hence, the purpose of the study was to describe the incidence and prevalence of glaucoma in the Danish population throughout a fifteen-year period. Furthermore, we wanted to investigate the geographic variation of glaucoma in Denmark and characterize the utilization pattern of glaucoma medication in the aforementioned period.

## Methods

### Population

The study population comprised all individuals living in Denmark in the period from 1996 to 2011. The government financed healthcare system requests all pharmacies in Denmark to register all prescriptions at an individual level by the so-called CPR-number. Likewise, the Danish National Registry of Patients has collected discharge diagnoses from public and private hospitals in Denmark since 1977. From 1995 the registry has used diagnoses as coded in the ICD-10.

Information on glaucoma medication was obtained from the Danish Registry of Medicinal Product Statistics. Following prescriptions were regarded as glaucoma medication (ATC-codes: S01ED01-05 = β-blockers; S01EE = prostaglandin analogs; S01EA = α2-adrenergic agonists, S01EB = parasympathomimetic drugs, S01EC = carbon anhydrase inhibitors; S01EA51, S01EB51, S01ED51 = fixed combination drugs).

Glaucoma was defined as a disorder treated as glaucoma. Patients were classified as “incident” by the time of their first prescription of glaucoma medication. Patients were categorized according to their number of treatment changes. A treatment change was defined as the start of a glaucoma treatment with more than 180-day time-wise gap between two purchases of glaucoma medicine.

### Statistics

Incidence rates were calculated in ten-year age strata. As previously mentioned, patients were classified as “incident” by the time of their first prescription of glaucoma medication. Incidence rate was defined as: new glaucoma cases, in each age strata, divided by the size of the Danish population (age strata). Prevalence was defined as: patients living with glaucoma, i.e., being treated with glaucoma medication, in each age strata, divided by the size of the Danish population, in each age strata.

The statistical analysis made use of the Poisson distribution and Two-proportion z-test.

All statistics were performed in SAS 9.2. P-values below 0.05 (two-sided) were considered significant. GIS Maps were performed in Arc Map 10.1.

### Ethics

The Danish Data Protection Agency approved the study (2007-58-0015, int. ref: GEH-2010-001). Retrospective register-based studies do not require ethical approval in Denmark.

## Results

A total of 116,592 incident glaucoma patients were identified in the period from 1996 to 2011, and 55% of the patients were females. The average age at onset was 66 (range: 0–105) years. Mean age for females was 68 years compared to 64 years for males. Baseline characteristics are shown in Table 1.

**Table 1:** Baseline characteristics of 116,592 glaucoma patients in the Danish population in the period from 1996 to 2011. The total Danish population, incidence and prevalence are calculated for each year.

	1996	1997	1998	1999	2000	2001	2002	2003	2004	2005	2006	2007	2008	2009	2010	2011
**Glaucoma**	** **	** **	** **	** **	** **	** **	** **	** **	** **	** **	** **	** **	** **	** **	** **	** **
New cases	7193	6986	7365	7192	6897	6700	6838	6987	6920	6736	7051	7379	7801	7738	8210	8599
Age ± SD	67±17	68±16	67±16	67±16	67±16	67±16	67±16	66±16	66±16	67±15	66±15	64±16	64±16	64±16	64±17	65±16
F (%)	55	57	56	56	55	56	55	55	55	55	55	55	55	54	53	54
M (%)	45	43	44	44	45	44	45	45	45	45	45	45	45	46	47	46
**Population**																
F (x 10^6^)	2.66	2.67	2.68	2.69	2.7	2.7	2.71	2.72	2.73	2.73	2.74	2.75	2.76	2.78	2.79	2.8
M (x 10^6^)	2.59	2.6	2.62	2.63	2.63	2.64	2.65	2.66	2.67	2.68	2.69	2.7	2.71	2.73	2.74	2.76
Total (x 10^6^)	5.25	5.28	5.29	5.31	5.33	5.35	5.37	5.38	5.40	5.41	5.43	5.45	5.48	5.51	5.53	5.56
**Incidence**																
F (‰)	1.55	1.51	1.54	1.5	1.41	1.39	1.39	1.42	1.4	1.35	1.42	1.47	1.55	1.49	1.56	1.64
M (‰)	1.19	1.14	1.24	1.2	1.18	1.11	1.16	1.17	1.16	1.13	1.17	1.24	1.3	1.31	1.41	1.45
Total (‰)	1.37	1.32	1.39	1.35	1.29	1.25	1.27	1.3	1.28	1.24	1.3	1.35	1.42	1.4	1.48	1.55
> 50 Y (%)	0.51	0.62	0.4	0.55	0.33	0.49	0.31	0.52	0.36	0.6	0.4	0.61	0.41	0.54	0.35	0.48
F > 50 Y (%)	0.55	0.71	0.34	0.55	0.24	0.42	0.2	0.5	0.28	0.68	0.36	0.7	0.37	0.53	0.25	0.39
M > 50 Y (%)	0.47	0.55	0.47	0.54	0.46	0.54	0.46	0.54	0.46	0.54	0.46	0.54	0.46	0.55	0.46	0.55
**Prevalence**																
F (%)	0.94	1.02	1.11	1.19	1.26	1.32	1.38	1.44	1.49	1.54	1.60	1.65	1.72	1.77	1.83	1.90
M (%)	0.65	0.72	0.79	0.86	0.92	0.97	1.03	1.08	1.13	1.18	1.23	1.28	1.34	1.40	1.47	1.54
Total (%)	0.79	0.87	0.95	1.03	1.09	1.15	1.2	1.26	1.32	1.36	1.42	1.47	1.53	1.58	1.65	1.72
> 50 Y (%)	2.09	2.23	2.38	2.5	2.62	2.72	2.82	2.92	3.02	3.12	3.22	3.32	3.42	3.51	3.63	3.76
F > 50 Y (%)	2.4	2.56	2.73	2.86	2.99	3.1	3.21	3.32	3.44	3.53	3.63	3.72	3.83	3.92	4.03	4.15
M > 50 Y (%)	1.73	1.85	1.97	2.08	2.19	2.27	2.37	2.46	2.56	2.66	2.76	2.86	2.96	3.06	3.18	3.32

Abbreviations: F = females; M = males; Y = years.

The majority of the cases (93.9%) were born in Denmark. The rest of the studied population was either from other Scandinavian countries (1.0%) or from countries outside of Scandinavia (5.0%). Data on nationality were missing in 0.1% of the cases.

### Incidence

**Fig 1:**
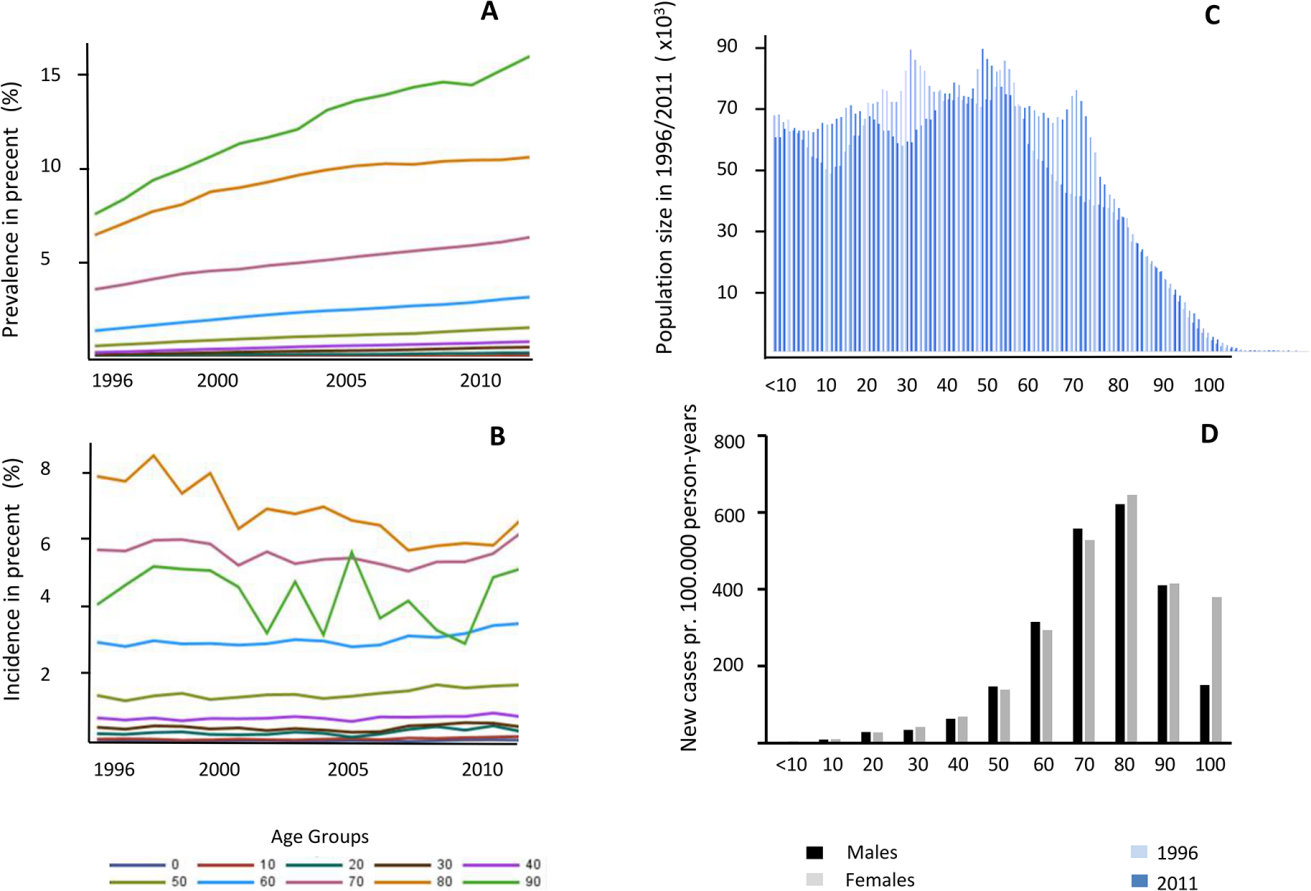
Prevalence, Incidence and age distribution of glaucoma patients in Denmark. A. Prevalence of glaucoma cases in Denmark in the period from 1996 to 2011. The percentage of female glaucoma patients above 50 years is shown in age intervals of 10 years. A significant increase in the prevalence throughout the period was found (*p* < 0.001). No significant difference is seen among females and males (*p* = 0.8). B. Incidence of glaucoma cases in Denmark in the period from 1996 to 2011. The percentage of female glaucoma patients above 50 years is shown in age intervals of 10 years. No significant difference is seen among females and males (*p* = 0.1). No significant change was observed in the incidence in the studied period. C. The age distribution in Denmark from 1996 and 2011. In 1996 the total population was 5.3 million and in 2011 5.6 million, respectively. Numbers are drawn from the Danish National Patient Registry. D. The age specific incidence rates of glaucoma in Denmark in the period from 1996 to 2011. Out of a total of 116,594 cases, the incident rates reveal a bell shaped distribution with a peak at the age of 80 years by 600 cases per 100,000 person-years.

The numeric incidence increased from 7200 to 8600 new glaucoma patients annually (*p* < 0.0001) in the period from 1996 to 2011. The increase was attributed to an aging population (Fig 1C, Table 1). Thus, the age specific incidence rate of glaucoma did not change during the study period (Fig 1D).

### Prevalence

A significant increase in the prevalence was seen in the period from 1996 to 2011. In 1996, the prevalence of glaucoma was 0.79% compared to 1.72% in 2011 (*p* < 0.0001). The prevalence was higher in females in the entire period (Table 1 and Table 2). However, the gender differences diminished during the period under investigation from a female to male ratio of 1.4 in 1996 to 1.2 in 2011 (Table 1). Glaucoma was more common in the elderly and affected approximately 10% of the population in the age group above 80 years, and as much as 15% in the population above 90 years (Fig 1, Table 2).

**Table 2:** Demographic prevalence of patients treated with glaucoma medication in 2011. Prevalence rates in 10 years age groups and the gender distribution are presented.

	Capital(M)	Capital(F)	Zealand(M)	Zealand(F)	South(M)	South (F)	Central(M)	Central(F)	North(M)	North (F)
Total pop.	838630	874994	405736	412585	598402	602963	631650	633951	291760	288533
Pop. > 50 Y	0.32	0.35	0.39	0.42	0.37	0.40	0.34	0.37	0.37	0.40
Pop. > 80 Y	0.03	0.05	0.03	0.05	0.03	0.06	0.03	0.05	0.03	0.06
Prevalence										
0–9 Y (%)	0.03	0.02	0.00	0.03	0.03	0.01	0.02	0.02	0.02	0.02
10–19 Y (%)	0.10	0.08	0.07	0.04	0.10	0.08	0.09	0.07	0.07	0.03
20–29 Y (%)	0.21	0.23	0.20	0.24	0.23	0.28	0.20	0.26	0.18	0.23
30–39 Y (%)	0.45	0.42	0.53	0.47	0.57	0.46	0.47	0.46	0.48	0.43
40–49 Y (%)	0.73	0.61	0.72	0.56	0.84	0.66	0.76	0.65	0.76	0.65
50–59 Y (%)	1.80	1.78	1.09	1.09	1.48	1.32	1.51	1.60	1.22	1.15
60–69 Y (%)	3.83	4.01	2.43	2.59	2.93	2.93	3.10	3.52	2.28	2.49
70–79 Y (%)	7.27	7.37	5.00	5.06	5.87	6.03	6.09	6.79	4.68	5.12
80–89 Y (%)	12.59	12.97	8.55	9.51	10.28	10.70	9.97	10.83	7.27	8.58
90–99 Y (%)	18.37	18.41	11.78	12.00	14.39	14.38	14.66	13.33	10.78	12.18
100–109 Y (%)	21.05	21.81	7.69	5.94	33.33	15.74	8.33	12.05	12.50	14.71

Abbreviations: Capital = The Capital Region of Denmark; Zealand, Region of Zealand; South = Region of Southern Denmark; Central = Region of Central Jutland; North = The North Denmark Region; M = Male; F = Female; pop = population; Y = years.

### Geographic differences

**Fig 2:**
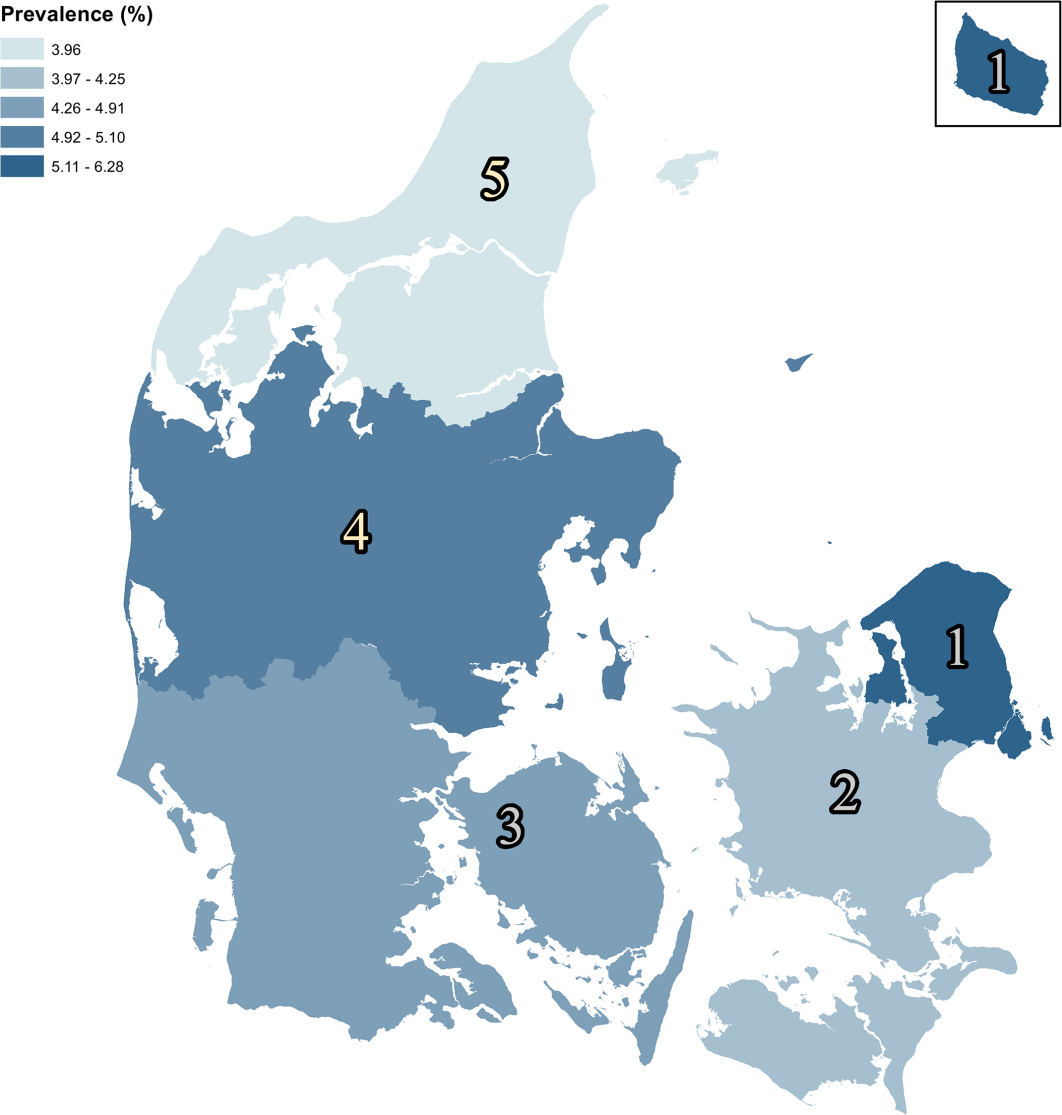
The demographic variation of glaucoma patients in Denmark. The map illustrates the differences in the prevalence of glaucoma cases in the age group from 50 to 60 years. The highest prevalence of glaucoma is found in the capital region of Denmark with a prevalence of 6.28%, which was significantly higher compared to the other regions (Table 3). 1) The Capital Region of Denmark, 2) Region of Zealand, 3) Region of Southern Denmark, 4) Region of Central Jutland, 5) The North Denmark Region.

The prevalence of glaucoma differed in the five regions of Denmark with a significantly higher number of cases in the capital region of Denmark. In this region the prevalence was 6.28% (95CI 6.21–6.36) compared to a prevalence of 3.96% (95CI 3.86–4.06) in the Northern part of Denmark (Fig 2, Table 3). Interestingly, these differences were unrelated to the geographical age-distributions (Table 2).

**Table 3:** The demographic variation of glaucoma in Denmark. The incidence and prevalence for 2011 is calculated.

Regions	Incidence	95% CI	Prevalence > 50 Y	95%CI
The Capital Region of Denmark	0.17	0.16–0.17	6.28	6.21–6.36
Region of Zealand	0.13	0.13–0.14	4.25	4.16–4.33
Region of Southern Denmark	0.17	0.16–0.17	4.91	4.83–4.98
Region of Central Jutland	0.14	0.13–0.14	5.10	5.02–5.18
The North Denmark Region	0.14	0.13–0.15	3.96	3.86–4.06

Abbreviations: Y = years

### Treatment pattern

**Fig 3:**
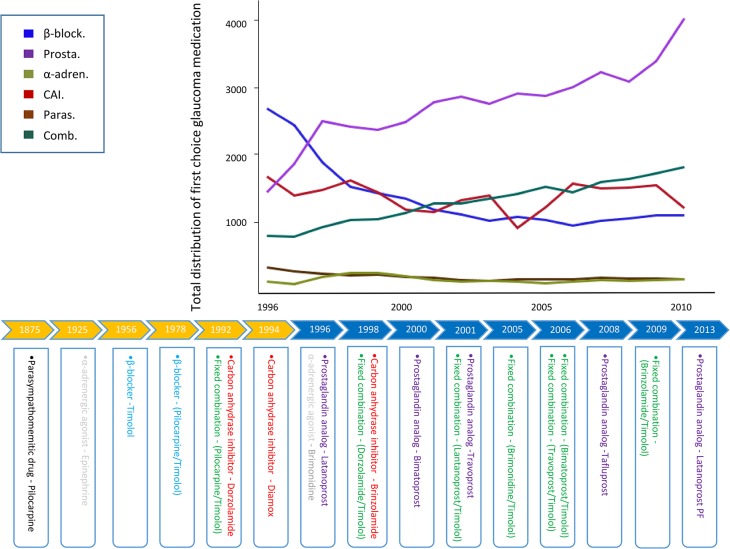
Total distribution of “first of choice” glaucoma medication in Denmark in the period from 1996 to 2011. The distribution of “first of choice” medication is shown throughout the study period. The use of β-blockers has decreased (*p* < 0.001), whereas the use of prostaglandin analogs and combination drugs has increased in the studied period (*p* < 0.001). The lower figure shows the time line of the introduction of glaucoma medication (modified from European Guidelines, 4^th^ edition). Abreviations: β-block., β-blockers; Prostagl., prostaglandin analogs; α-adren., α2-adrenergic agonists; Paras., parasympathomimetic drugs; CAI, carbon anhydrase inhibitors; Comb., combination drugs.

The “First drug of choice” changed from β-blockers in 1996 to prostaglandin analogs in 2011 (Fig 3, Table 4). In 2011 47% used prostaglandin analogs as mono therapy, whereas only 13% were treated with β-blockers. Over all, prostaglandin analogs and fixed combination drugs increased between 1996 and 2011 (*p* < 0.001), β-blockers, carbonic anhydrase inhibitors and parasympathomimetic drugs decreased (*p* < 0.001), whereas the use of α2-adrenergic agonists were stable (Fig 3, Table 4).

**Table 4:** Baseline characteristics of glaucoma medication in the Danish population in the period from 1996 to 2011.

Characteristic	Total	1996	1997	1998	1999	2000	2001	2002	2003	2004	2005	2006	2007	2008	2009	2010	2011	1996 vs. 2011(p-value)
**First choice**																		
β-blockers	22362	2708	2470	1912	1551	1457	1374	1215	1141	1044	1099	1054	978	1040	1084	1131	1124	
%	19	38	35	26	22	21	21	18	16	15	16	15	13	13	14	14	13	<0.001
Prostagl.	44337	1466	1891	2523	2441	2387	2514	2795	2886	2780	2933	2893	3023	3249	3108	3411	4041	
%	38	20	27	34	34	35	38	41	41	40	44	41	41	42	40	42	47	<0.001
α2-agonists	2774	140	107	211	269	275	229	162	141	154	144	125	142	170	154	171	182	
%	2	2	2	3	4	4	3	2	2	2	2	2	2	2	2	2	2	0.5
CAI	22478	1699	1415	1502	1637	1460	1207	1169	1346	1414	941	1246	1590	1525	1537	1567	1230	
%	19	24	20	20	23	21	18	17	19	20	14	18	22	20	20	19	14	<0.001
Parasymp.	3423	354	290	265	241	248	217	198	166	154	179	180	174	204	187	186	180	
%	3	5	4	4	3	4	3	3	2	2	3	3	2	3	2	2	2	<0.001
Combined	21178	826	813	952	1053	1070	1159	1299	1307	1374	1440	1553	1472	1613	1668	1744	1842	
%	18	11	12	13	15	16	17	19	19	20	21	22	20	21	22	21	21	<0.001
**Prescription no**																		
1. presc.	80633	4056	3991	4573	4546	4373	4135	4470	4621	4666	4500	4865	5381	5935	6095	6815	7611	.
%	76	66	67	72	72	72	71	73	73	75	74	75	79	81	83	87	91	<0.001
2. presc.	17894	1234	1249	1093	1121	1074	1146	1179	1216	1122	1175	1242	1135	1160	1072	956	720	
%	17	20	21	17	18	18	20	19	19	18	19	19	17	16	15	12	9	<0.001
3. presc.	5538	594	506	471	460	430	446	403	380	380	345	330	273	233	163	92	32	
%	5	10	8	7	7	7	8	7	6	6	6	5	4	3	2	1	0	<0.001
4. presc.	1442	216	203	153	155	144	114	83	94	78	77	60	36	19	8	2	0	
%	1	4	3	2	2	2	2	1	1	1	1	1	1	0	0	0	0	<0.001
5. presc.	282	59	47	45	25	32	24	18	11	11	5	1	3	1	0	0	0	
%	0	1	1	1	0	1	0	0	0	0	0	0	0	0	0	0	0	<0.001

Abbreviations: Prostagl. = prostaglandin analogs; Parasymp. = parasympathomimetic drugs; CAI = carbon anhydrase inhibitors; Combined = combination drugs.

### The pattern of combined medication in the period from 1996 to 2011

During the study period 23% of glaucoma patients were treated with two or more glaucoma medications. The second choice glaucoma medication was often fixed combination drugs. Patients treated with two separate medications decreased from 20% to 9% throughout the investigated period (*p* < 0.001). Moreover, patients treated with more than two glaucoma drugs decreased from 15% to 0.4% (*p* < 0.001) (Table 4). Overall, the pattern of glaucoma treatment revealed a preference of prostaglandin analogs as “first drug of choice” and a switch to a combination drug as second choice (Table 4).

## Discussion

The present study demonstrated an increasing number of people with glaucoma in the Danish population in the period from 1996 to 2011. We found a total prevalence of glaucoma of 1.72% and a prevalence of 3.76% in the population above 50 years. In comparison, previous estimations of the prevalence of glaucoma in Denmark have been significantly lower[9,10].

Until 1996 official medical statistics were published in Denmark. In these reports the wholesale of registered pharmaceutical agents were given as “Defined Daily Dosages” per 1000 inhabitants/year. Based on these numbers the total prevalence of glaucoma patients in Denmark was estimated to 0.33% in 1989[9]. A later study estimated the Danish prevalence of glaucoma based on questionnaires carried out among Danish Eye Practitioners [10]. Lundberg et al. reported an overall prevalence on 0.65% well knowing that their study underestimated the true prevalence. A clear correlation between glaucoma and age was shown. In this matter the prevalence was shown to increase from 0.5% to 5% in the population at 50 and 80 years, respectively[10].

A recent meta-analysis estimated the prevalence of glaucoma worldwide and revealed a global prevalence of glaucoma of 3.54% in the population between 40 and 80 years[12]. The prevalence in Europe was shown to be 2.51[12].

In the present study the estimation of glaucoma cases in Denmark was based on numbers from the Danish Registry of Medicinal Product Statistics, which allows a more accurate estimation of persons that use glaucoma medication.

Obviously, estimates of a prevalence based on consumption of glaucoma medication are still associated with considerable inaccuracy. Hence, it is generally accepted that a great number of glaucoma cases remain undiagnosed. Unrecognized glaucoma subjects are either a result of ophthalmologists missing the diagnosis or patients not presenting to their ophthalmologist. Several studies have indicated that around 50% of OAG cases are unrecognized[13–16].

On the other hand over prescription may also bias our results. In this matter it is not uncommon to find prescriptions for glaucoma drugs in patients with no disc and visual field changes. In addition, treatment with glaucoma medication is often seen in patients with a history of the highest IOP of less than 25 mmHg. In these scenarios the ophthalmologists overestimate the benefit of therapy, while underestimating the associated risk of treatment[17]. Bearing in mind the aforementioned limitations, we however believe that the cases extracted from National Prescription Registry lead to a reliable estimate of the prevalence of glaucoma in Denmark.

Previous studies have shown an increased prevalence of glaucoma in urban areas compared to rural areas[12]. In the present study the demographic variation of glaucoma cases was estimated in the five existing regions of Denmark. We found a significant higher prevalence of glaucoma in the capital region of Denmark compared to other regions (Fig 2, Table 3). In support of this regional difference in Denmark, it has previously been shown that the highest number of glaucoma prevalence was found in Copenhagen followed by the second largest city in Denmark, Aarhus[9]. Since the higher prevalence of glaucoma was not corresponding to a higher number of elderly (Table 2), the significant regional differences observed in this study, may reflect a more readily available healthcare in urban areas. In addition, the population in rural areas of Denmark may have a tendency to neglect their eye problems, which consequently leads to an increased number of unrecognized glaucoma cases.

Due to reimbursement from the national healthcare system, all Danish pharmacies have been obligated to register all prescriptions dispensed to the Danish Registry of Medicinal Product Statistics since 1996. In this way an ensured and complete registration, without selection bias, allows a detailed analysis of distributed glaucoma medication. In comparison, the use of data based on ICD-10 coding in the National Registry of Patients will underestimate the number of glaucoma cases due to errors or lack of registration by general ophthalmologists. In the same matter, surgical procedures and laser treatments for glaucoma will be under-estimated if using ICD-10 coding. Therefore, an estimation of such procedures will require questionnaire studies and is beyond the scope of the present study.

Here we investigated the pattern of glaucoma medication in the period from 1996 to 2011 (Fig 3, Table 4). Among the 116,552 identified glaucoma cases the most used mono therapy changed from β-blockers to prostaglandin analogs in the study period. The change can be explained by the introduction of prostaglandin analogs in 1996 as well as a higher pressure-lowering efficacy of prostaglandin analogs compared to β-blockers[18]. A decrease in the use of carbon anhydrase inhibitors and parasympathomimetic drugs was found throughout the studied period, whereas the use of α2-adrenergic agonists remained consistent. Over all, the use of several simultaneous glaucoma medications decreased throughout the study period (Table 4). Increased awareness of adherence challenges might explain this decrease. Furthermore, the continuous introduction of combined medications and the introduction of selective laser treatment and new surgical procedures may also explain the reduced number of patients receiving multiple glaucoma medications (Table 4). In support of this assumption, the prescription of combination drugs increased significantly throughout the study period (Fig 3, Table 4). In comparison to the Danish prescription patterns, recent studies investigated the use of glaucoma medication in the American and the British population, respectively[19,20]. A similar treatment pattern was found. Both studies found an increased use of prostaglandin analogs along with a decreased use of β-blockers. The only significant difference from the current study was an increased use of α2-adrenergic agonists among the American population[19]. The lack of increase in α2-adrenergic agonist treatment in Denmark may be due to the unavailability of the α2-adrenergic agonist suspension, Alphagan P, which has been shown to cause significantly fewer cases of allergy compared to the European suspension[19,21].

## Conclusion

To our knowledge the present study is the largest nationwide study ever made and must be a close-to-real-life-picture of the utilization of glaucoma medication on a nationwide scale. The age specific incidence rate of glaucoma cases was shown to be constant, and the increased prevalence correlates with an aging population. Compared to previous studies the present study reveals a higher incidence and prevalence than previously estimated. Hence, as many as 3.76% of citizens above 50 years suffer from glaucoma, and as many as 10% of citizens above 80 years are treated with glaucoma medication. Increasing evidence implies that systemic diseases might be associated with glaucoma. Based on the strength of the complete data sample from an entire population future studies will investigate possible comorbidities with glaucoma, and hopefully contribute to a better understanding of the management of glaucoma.
